# Effectiveness of Pulse Intravenous Infusion of Methylprednisolone on Pain in Patients with Lumbar Disc Herniation: A Randomized Controlled Trial

**DOI:** 10.5812/aapm-149442

**Published:** 2024-09-07

**Authors:** Hassan Reza Mohammadi, Yousef Asadoola, Ali Erfani, Nazila Ghoreishi Amin, Hosein Karimiyarandi, Sohrab Sadeghi, Mohammad Abiri

**Affiliations:** 1 Department of Neurosurgery, School of Medicine, Imam Hossein Hospital, Shahid Beheshti University of Medical Sciences, Tehran, Iran; 2 Department of Nursing, Al-Kut University College, Wasit, Iraq; 3 Department of Radiology, Keck School of Medicine, University of Southern California (USC), Los Angeles, California, USA; 4 School of Medicine, Emam Khomeini Hospital, Ilam University of Medical Sciences, Ilam, Iran; 5 Ilam University of Medical Sciences, Ilam, Iran

**Keywords:** Pain, Methylprednisolone, Lumbar Disc Herniation, Randomized Controlled Trial

## Abstract

**Background:**

Lumbar disc herniation (LDH) can cause pain in the lower back and leg, as well as numbness or weakness in the affected area. Various steroids, including methylprednisolone, are currently used for treatment.

**Objectives:**

This study aimed to compare the effectiveness of pulse intravenous infusion of 500 mg methylprednisolone with common non-steroidal anti-inflammatory drugs (NSAIDs) in relieving pain and improving the clinical condition of patients with lumbar disc herniation.

**Methods:**

This clinical trial, registered under code IRCT20211116053077N1, included an experimental group (37 patients) and a control group (35 patients). Pain assessments were conducted before treatment, and at one, two, and three weeks, as well as one and six months after treatment. The control group received common painkillers (diclofenac sodium tablets 100 mg), while the experimental group received a single dose of 500 mg methylprednisolone sodium succinate (intravenous injection in 500 cc normal saline). Pain scores were analyzed using SPSS 16 and statistical tests such as ANOVA, independent *t*-tests, and repeated measures ANOVA.

**Results:**

Prior to intervention, the mean (SD) pain score was 8.7 (3.57) in the experimental group and 8.17 (0.66) in the control group (P > 0.76). Six months after methylprednisolone injection, the mean (SD) pain score in the experimental group was 1.56 (0.83), compared to 6.48 (0.91) in the control group (P = 0.000). Analysis of variance indicated that methylprednisolone significantly reduced pain in patients with LDH (P = 0.000, F = 660.668).

**Conclusions:**

Given the effectiveness of intravenous pulse infusion of 500 mg methylprednisolone compared to common NSAIDs in relieving pain and improving clinical outcomes for patients with lumbar disc herniation, the use of this drug is recommended for pain reduction in these patients.

## 1. Background

Back pain is a leading cause of absenteeism and disability in the workplace and is a major reason for hospitalization. The global cost of back pain for an individual is estimated at around $100 million (1, 2). Patients often visit neurosurgery clinics for back pain, which may be managed through outpatient care, physical therapy, narcotic drugs, non-steroidal anti-inflammatory drugs (NSAIDs), or surgery (3, 4). Lumbar disc herniation (LDH) is one of the most prevalent degenerative spine diseases, with a reported prevalence of 2 - 3% (5). Recently, the incidence of LDH has increased, especially among younger people, likely due to decreased physical activity and weight gain. The highest prevalence is reported among individuals aged 30 to 50 (6-8).

The lumbar spine comprises vertebrae and intervertebral discs located in the lower back. Lumbar disc herniation can stimulate or compress adjacent nerves, leading to pain and other symptoms (9-11). Damage to the intervertebral discs in the lower lumbar region, particularly in the L4-L5 or L5-S1 discs, often results from the high mobility of the lower lumbar area (12, 13).

Lumbar disc herniation related pain may manifest in the lower back, leg, and can also include numbness or weakness in the affected area (9-11). Symptoms of lumbar disc herniation include back pain, leg pain, radiating pain along the sciatic nerve, and abnormal gait (14). In the initial stage of the disease, patients primarily experience back pain, while later stages are marked by leg pain and radiating pain (7, 15). Untreated LDH pain can significantly impair quality of life and place a caregiving burden on patients’ caregivers. Recently, there has been an increase in the percentage of patients undergoing surgery for lumbar disc herniation (16, 17).

Lumbar disc herniation is defined through various imaging and intraoperative pathology classifications, which are evaluated using different methods (17). For cases that develop, treatment recommendations during the first 4 to 6 weeks after symptom onset typically include rest and therapeutic measures as advised by a physician. If symptoms persist beyond this period and are confirmed by clinical findings and MRI, surgical treatment may be suggested (2, 6).

Pain is a major concern for LDH patients, with effects extending beyond physical discomfort to include psychological issues, decreased quality of life, and socio-economic impacts (18-22). Corticosteroids, which have anti-inflammatory properties and varying mechanisms of action, are used to reduce pain. These steroids are categorized based on their duration of effect into short, medium, and long-acting types. Anti-inflammatory doses of steroids are commonly employed in the initial treatment phase for various rheumatic diseases (23-25). Currently, steroids are available in various forms, including topical, local injections, and intravenous administration. Methylprednisolone is one such steroid (23-25).

## 2. Objectives

This study aims to compare the effectiveness of pulse intravenous infusion of 500 mg methylprednisolone with common non-steroidal pain relievers in relieving pain and improving the clinical condition of patients with lumbar disc herniation.

## 3. Methods

This clinical trial, with ethics code IR.MEDILAM.REC.1400.152 and clinical trial code IRCT20211116053077N1, included experimental (45 patients) and control (45 patients) groups. The study involved individuals over 18 years of age with evident lumbar herniation on MRI results. Participants experienced severe back pain for at least 6 months, with pain extending to the lower limbs.

Exclusion criteria included individuals under 18 years of age, those with a history of spine surgery, patients with neurological defects such as plegia, and those who were unavailable for follow-up (e.g., failure to follow up, death, relocation, or inability to return). Additionally, patients or their families needed to have a mobile phone for communication. Non-cooperation throughout the study (from the beginning to 6 months later, when completing the final questionnaire) also led to exclusion.

Patients were randomly assigned to either the experimental or control group using random blocks. They were given cards and randomly selected one to determine their group assignment.

The pain level was assessed using a scale from 0 to 10 (26). Measurements were taken before treatment and at one, two, and three weeks after treatment, as well as at one and six months after treatment. The control group received common painkillers (diclofenac sodium tablets 100 mg), while the experimental group received methylprednisolone sodium succinate 500 mg (one dose administered intravenously in 500 cc of normal saline). All patients rested for 24 hours post-injection, and MRI scans were conducted before and six months after the intervention to compare and interpret the results.

Ethical considerations included obtaining written consent from participants, randomly assigning study groups, providing free interventions (visits, drugs, MRIs) for both groups, and ensuring patient confidentiality. Pain scores were analyzed using SPSS 16, with analytical tests such as ANOVA, independent *t*-tests, and repeated measures ANOVA.

## 4. Results

Of the 90 patients initially included in the study, 72 patients (35 in the control group and 37 in the experimental group) were included in the analysis stage. Exclusions occurred due to reasons such as death, surgery, lack of follow-up, and withdrawal of consent to participate in the study.

According to Table 1, there were no significant differences between the demographic characteristics, including age, gender, and marital status (P > 0.05). Before the intervention, the mean (SD) pain score was 8.7 (3.57) in the experimental group and 8.17 (0.66) in the control group (P > 0.76). Six months after the methylprednisolone injection, the mean (SD) pain score in the experimental group was 1.56 (0.83), compared to 6.48 (0.91) in the control group (P = 0.000) (Table 2). 

**Table 1.** Comparison of Demographic Characteristics of Patients Under Study ^a^

Measurement time	Experimental group	Control Group
**Age**	50.29 ± 8.09	49.68 ± 9.38
**Gender**		
Man	25 ± 67.6	24 ± 68.6
Female	12 ± 32.4	11 ± 31.4
**Marital status**		
Married	27 ± 73	23 ± 65.7
Single	10 ± 27	12 ± 34.3

^a^ Values are expressed as Mean ± SD.

**Table 2.** Comparison of Pain Intensity of Patients Before and After the Intervention ^a^

Measurement Time	Experimental Group	Control Group	P-Value
**Before intervention**	8.70 ± 3.57	8.17 ± 0.66	0.76
**One week after the intervention**	2.64 ± 1.43	7.85 ± 0.84	0.48
**Two weeks after the intervention**	2.18 ± 1.54	7.37 ± 1.05	0.05
**Three weeks after the intervention**	2.43 ± 1.3	7.25 ± 0.85	0.000
**One month after the intervention**	1.35 ± 0.82	7.17 ± 1.04	0.000
**6 months after the intervention**	1.56 ± 0.83	6.48 ± 0.91	0.000

^a^ Values are expressed as Mean ± SD.

The results of the analysis of variance indicated that methylprednisolone significantly reduces pain in patients with LDH (P = 0.000, F = 660.668). According to Table 3, Mauchly's Test of Sphericity for pain showed Mauchly's W value of 0.25 with a significance level of 0.000 (Tables 3 and 4). 

**Table 3.** Mauchly's Test of Sphericity for Pain

Within Subjects Effect	Mauchly's W	Approx. Chi-Square	Sig.	Epsilon		
				Greenhouse-Geisser	Huynh-Feldt	Lower-Bound
**Factor 1**	0.025	125.383	0.000	0.413	0.439	0.200

**Table 4.** Correlated One-Way ANOVA Measured Four Times After Follow Up Tests

Source	Type III Sum of Squares	df	Mean Square	F	Sig.
**Intercept**	2200.905	1	2200.905	660.668	0.000
**Residual Error**	119.928	36	3.331		

## 5. Discussion

Throughout life, individuals may encounter various physical problems, including pain, which can significantly impact all dimensions of health (27-32). To alleviate this pain, both pharmacological and non-pharmacological methods are available (33-35). One common approach involves using corticosteroids, which are effective in reducing pain due to their ability to suppress cytokines (36).

The findings indicate that methylprednisolone, a corticosteroid, effectively reduces patient pain. Numerous pharmacological and non-pharmacological interventions have been explored to manage pain in patients with lumbar disc herniation (LDH). Among non-pharmacological interventions, acupuncture has been noted for its effectiveness. For example, Zhang et al. conducted a meta-analysis of 10 studies involving 838 patients, finding that acupuncture significantly reduces pain in LDH patients (22). Similarly, Kwon et al. studied the impact of lumbar motion-style acupuncture on back pain resulting from road accidents. Their study showed that pain intensity in the experimental group decreased from 6.7 to 3.7, while in the control group, it decreased from 6.3 to 5.55, suggesting that the intervention was effective in reducing pain (37). Acupuncture is a therapeutic or preventive intervention involving the insertion of needles into specific acupoints to enhance patient health (38, 39).

Various studies have investigated the impact of corticosteroids on patients' health. For instance, Stone et al. demonstrated that corticosteroids effectively reduced patient pain (40). Iranmanesh et al. found that corticosteroids alleviated pain in patients with root canal conditions (41). Hayward et al. reviewed eight studies involving 743 patients, revealing that corticosteroids significantly reduced sore throat when administered for more than 6 hours (42). Additionally, Kullenberg et al. showed that corticosteroids improved pain and function in elderly patients with knee osteoarthritis (43). Corticosteroids are utilized both as primary and adjunctive pain relievers, playing a crucial role in reducing skeletal and muscular pain (25).

In relation to low back pain, Friedman et al. examined 82 patients with radicular low back pain and found that corticosteroids effectively reduced their pain (44). Similarly, Quraishi's meta-analysis of three IRCT studies confirmed that corticosteroids alleviated pain in lumbar radiculopathy patients (45), aligning with the findings of this study.

### 5.1. Conclusions

Given the effectiveness of intravenous pulse infusion of methylprednisolone 500 mg compared to common non-steroidal pain relievers in alleviating pain and improving the clinical condition of patients with lumbar disc herniation, it is recommended to use this drug to reduce patient pain.

## Data Availability

The dataset presented in the study is available on request from the corresponding author during submission or after publication.

## Clinical Trial Registration Code:

IRCT20211116053077N1​.
